# Supplementary material for: Predictive Limitations of the Geriatric Trauma Outcome Score: A Retrospective Analysis of Mortality in Elderly Patients with Multiple Traumas and Severe Traumatic Brain Injury
Source: Diagnostics (Basel). 2025 Feb 28;15(5):586. doi: 10.3390/diagnostics15050586 (PMC11899710; doi:10.3390/diagnostics15050586)

Supplementary Table S1. Geriatric Trauma Outcome Score (GTOS), observed and predicted mortality, and model performance according to head AIS severity in elderly patients with multiple traumas and TBI

Group	n	GTOS	Observed in-hospital mortality, % (n)	GTOS predicted risk of mortality, %	AUC	95% CI
**All Patients**	1283	126.9±31.9	17.9 (230)	17.6	0.798	0.770-0.825
**Head AIS ≥ 1**	797	134.1±32.7	22.8 (182)	21.2	0.769	0.732-0.804
**Head AIS ≥ 2**	778	135.2±32.2	23.2 (181)	21.6	0.766	0.730-0.802
**Head AIS ≥ 3**	662	138.3±31.9	26.0 (172)	23.7	0.743	0.703-0.783
**Head AIS ≥ 4**	390	149.6±29.4	39.5 (154)	28.8	0.657	0.603-0.710
**Head AIS ≥ 5**	232	160.3±27.3	50.9 (118)	35.3	0.501	0.428-0.578

Supplementary Figure S1. ROC curves for Geriatric Trauma Outcome Score (GTOS) and modified GTOS for predicted in-hospital mortality in patients with multiple traumas and severe TBI

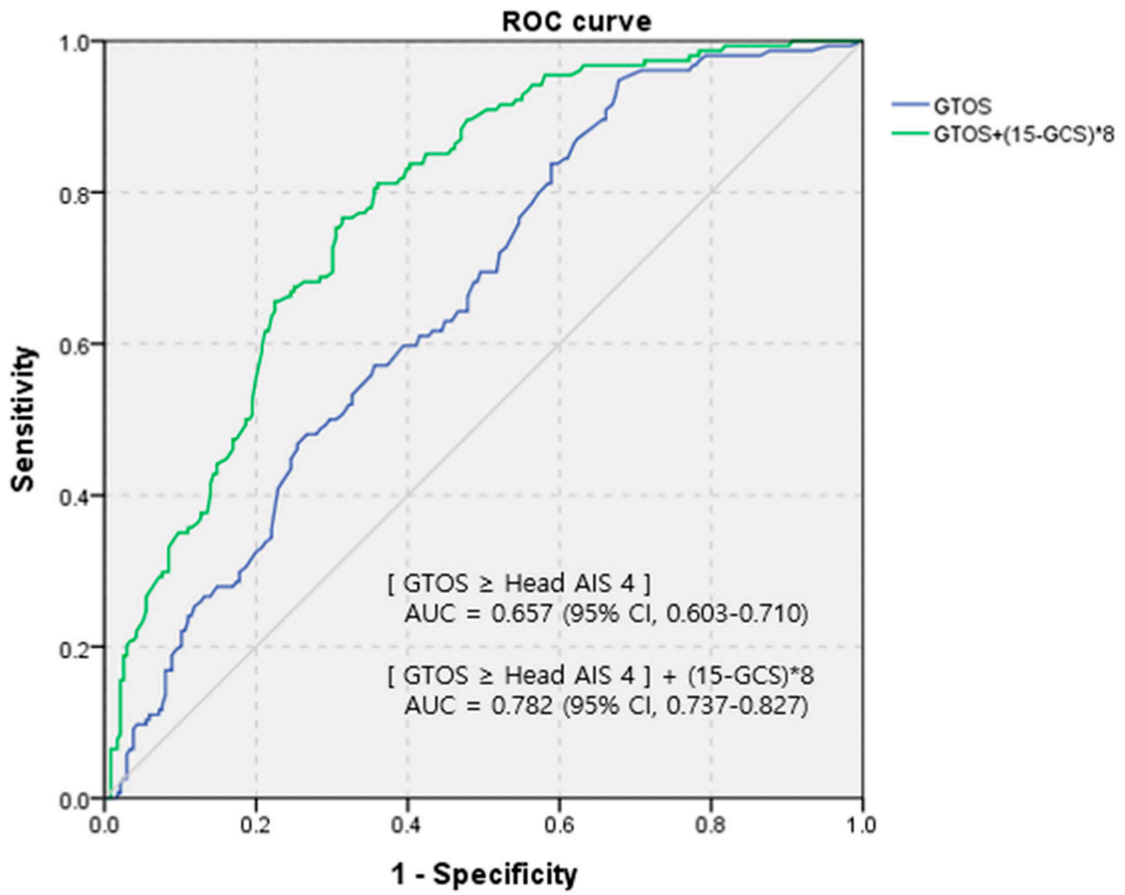

Supplement: Supplementary file 1 [file diagnostics-15-00586-s001.zip › diagnostics-3467794-supplementary.pdf]
